# Neutralization of IL-4 and IFN-γ Facilitates inducing TGF-β-induced CD4^+^Foxp3^+^ Regulatory Cells

**Published:** 2008-03

**Authors:** Xiaojuan Tao, Jilin Ma, Yonghua Zhang, Jianning Yu, Long Cai, Juhua Wang, Song Guo Zheng

**Affiliations:** 1 *Division of Rheumatology, Immunology and Nephrology, Zhejiang Traditional Chinese Medicine and Western Medicine Hospital, Hangzhou, P.R. China;*; 2 *Division of Rheumatology and Immunology, Department of Medicine, University of Southern California, Keck School of Medicine, Los Angeles, USA*

**Keywords:** suppressor/regulatory T cells, cytokine, Foxp3, autoimmunity

## Abstract

It has been well recognized that TGF-β is able to induce CD4^+^CD25^+^Foxp3^+^ suppressor/regulatory T (iTreg) cells and IL-2 facilitates iTreg induction and expansion, however, only half of TGF-β-induced CD4^+^CD25^+^ cells express Foxp3 and remaining CD4^+^CD25^+^Foxp3- cells may represent effector cells. Whether other factor(s) can increase Foxp3 expression by CD4^+^CD25^+^ cells induced with TGF-β is still unclear. Here we show that addition of exogenous IFN-γ or IL-4 diminished the ability of TGF-β to induce Foxp3 expression and IL-2 failed to rescue this decreased Foxp3 expression. Conversely, neutralization of IFN-γ and IL-4 significantly enhanced the ability of TGF-β to induce Foxp3 and develop the suppressive activity, indicating that different cytokine profiles affect the differentiation of CD4^+^CD25^+^Foxp3^+^ subset induced by TGF-β. These results show that combination of antibodies against IFN-γ and IL-4 and TGF-β enhances the efficacy of generation and function of iTreg cells and may therefore provide a novel therapeutic strategy for the treatment of autoimmune and other chronic inflammatory diseases.

## INTRODUCTION

Naturally occurring, thymus-derived CD4^+^CD25^+^ suppressor/regulator T cells (nTregs) play a pivotal role in maintenance of immune tolerance to self antigens (1, 2). Lack or dysfunction of nTregs have appeared to be involved in the development and progression of autoimmune diseases, such as type I diabetes, multiple sclerosis, rheumatoid arthritis and active lupus (3-6). nTregs account for only 5%-10% of peripheral CD4^+^ T cells in mice and 1-2% in human (7). Foxp3, one of forkhead family transcription factor members, is a lineage specification factor for Tregs and plays an important role in the development and function of Treg cells (8, 9). It is likely that manipulation of nTreg cells may provide another approach to treat autoimmune diseases. Nonetheless, small numbers and decreased suppressive activity following expansion limit their potential for therapeutic considerations (10).

Treg cells are heterogeneous and composed of either thymus-derived nTregs or those that can be induced outside of the thymus (induced Treg, iTreg) (10). Although several CD4^+^ iTreg cell subsets have been reported, the transforming growth factor-β (TGF-β)-induced iTreg subset has attracted attention since it has properties similar to that of nTreg (11-16). TGF-β is able to induce TCR engaged CD4^+^CD25^-^Foxp3^-^ precursor T cells to become CD4^+^CD25^+^ cells that express Foxp3^+^ and appear to become suppressor or regulatory T cells *in vitro* and *in vivo* (11-21).

TGF-β is a pleiotropic cytokine exerting a differential impact on the differentiation of T lymphocytes depending on the target cell type and distinct cytokine milieu (22). Whereas TGF-β induces the differentiation of Foxp3^+^ Treg cells in the presence of IL-2 (23, 24), TGF-β also facilitates the induction of IL-17-producing (Th17) cells, at least in animal models (25-27). In addition, TGF-β has a critical function as an antagonist of Th1 development affecting IFN-γ as well as T-bet (28, 29), of Th2 differentiation affecting IL-4 (30, 31). Although it has been reported that non-T cell-derived IL-6 abolishes the ability of TGF-β to induce Foxp3^+^ cells (32), it is still unclear whether other Th1 and Th2 cytokines produced by T cells also affect the differentiation of iTreg cells induced by TGF-β.

In the present work, we confirmed that TGF-β is able to induce naïve CD4^+^CD25^-^ cells to express Foxp3 and develop suppressive activity in the absence of antigen presenting cells (APC). However, the addition of exogenous IFN-γ or IL-4 diminished the ability of TGF-β to induce Foxp3 expression. Interestingly, neutralization of IFN-γ and IL-4 significantly enhanced the ability of TGF-β to induce Foxp3 and develop the suppressive activity. Thus, the combination of TGF-β and neutralization of IFN-γ and IL-4 may provide a new protocol for the generation of TGF-β-induced iTreg cells *ex vivo*.

## MATERIALS AND METHODS

### Mice

C57BL/6 mice were purchased from The Jackson Laboratory and Shanghai Animal Institute, respectively. Mice used in all experiments were 8–12 weeks of age. All animals were treated according to National Institutes of Health guidelines for the use of experimental animals with the approval of the University of Southern California Committee for the Use and Care of Animals and Natural Science Foundation of China guidelines for the use of animals with approval of Committee for the Use and Care of Animals from Zhejiang Traditional Chinese Medicine and Western Medicine Hospital, P. R. China.

### Reagents

PE-, FITC-, or CyChrome-conjugated anti-CD8 (53-6.7), CD11b (M1/70), B220 (RA36B2), CD62L (MEL-14), and respective matched control IgG Abs were purchased from BD Pharmingen. Conjugated anti-CD3 (17A2), CD4 (GK1.5), CD25 (PC61.5) and matched control IgG Abs was obtained from eBioscience. The FITC-conjugated anti-mouse/rat Foxp3 staining kit (FJK-16s) was purchased from eBioscience. TGF-beta1, IFN-γ, IL-4 and rhIL-2 were purchased from R&D Systems. Unconjugated anti-IL-4 and anti-IFN-γ were purchased from BD Pharmingen. Anti-CD3 and anti-CD28 antibodies were also purchased from BD Pharmingen. TRIzol was purchased from Invitrogen Life Technologies. AIM-V serum-free medium (Invitrogen Life Technologies) supplemented with 100 U/ml penicillin, 100 μg/ml streptomycin, and 10 mM HEPES (all obtained from Invitrogen Life Technologies) was used for the generation of CD4^+^ iTreg or control cells. RPMI 1640 medium supplemented as just described with 10% heat-inactivated FCS (HyClone Laboratories) was used for all other cultures.

### Cell Purification

T cells were prepared from spleen cells by collecting nylon wool column non-adherent cells as described previously (17). CD4^+^ T cells were isolated by magnetic bead-based negative selection. In brief, T cells were labeled with PE-conjugated anti-CD8, anti-CD11b, and anti-B220 mAbs, incubated with anti-PE magnetic beads, and loaded onto MACS separation columns (Miltenyi Biotec). CD4^+^ cells were further labeled with FITC-conjugated anti-CD25 mAb, and CD4^+^CD25^+^ and CD4^+^CD25^-^ cells were obtained by cell sorting (cell purity of CD4^+^CD25^-^ cells is >98%). To prepare naïve CD4^+^CD25^-^ cells, CD4^+^CD25^-^ cells were labeled with PE-conjugated anti-CD62L and positively selected by anti-PE magnetic beads.

### *In vitro* T cell stimulation

Naïve CD4^+^CD25^-^ T cells were stimulated with immobilized anti-CD3 (0.5 μg/ml), soluble anti-CD28 (1 μg/ml) and IL-2 (20 u/ml) ± TGF-β (2 ng/ml) in the presence or absence of exogenous IFN-γ (20 ng/ml) or IL-4 (20 ng/ml), anti-IFN-γ (10 μg/ml) and anti-IL-4 (10 μg/ml) or control rat IgG1 (10 μg/ml) for 4 days.

### Flow cytometry analysis and intracellular cytokine staining

Prior to staining, cells were washed and re-suspended in staining buffer containing 1x PBS, 2% BSA, 10mM EDTA and 0.01% NaN3, To block non-specific staining, anti-CD16/32 antibody (2.4G2) was added. Antibodies for cell surface markers were added and cells were incubated 25 min on ice. Following staining, the cells were washed twice and analyzed the same day or fixed in PBS containing 1% paraformaldehyde and 0.01% NaN3, and cells were examined on the Epics XL-MC and data analyzed using EXPO32 software. Intracellular Foxp3 staining was performed as per Foxp3-staining kit protocol.

### *In vitro* proliferation/suppression assays

Proliferation assays were performed by stimulating responding T cells in 96 flat-bottom microtiter plates in RPMI 1640 with immobilized anti-CD3 (0.5 μg/ml), soluble anti-CD28 for 72 h at 37°C in 5% CO2. For suppression assays, TGF-beta1-treated or untreated T cells were co-cultured with CD4^+^CD25^-^ responder T cells with immobilized anti-CD3 (0.5 μg/ml), soluble anti-CD28 in 96-well plates for 72 h at 37°C/5% CO2. Cell cultures were pulsed with 1 uCi ^3^H-thymidine for the last 16 h to determine the extent of suppression.

### RT-PCR for Foxp3 expression

Total RNA was extracted from cells using TRIzol reagent and used to determine the expression and relative level of the transcription factor Foxp3. First-strand cDNA was synthesized using Omniscript TR kit (Qiagen) with random hexamer primers (Invitrogen Life Technologies). Foxp3 and hypoxanthine guanine phosphoribosyl transferase (HPRT) mRNA was measured by a semiquantitative RT-PCR using published primers (8). The relative expression of Foxp3 was determined by normalizing expression of each target to HPRT.

### Statistical analysis

Results are expressed as mean ± SEM, and are representative of 3-5 similar experiments. Analysis for statistically significant differences was performed with Student’s t-test. *P*<0.05 was considered a difference, and *P*<0.01 was considered a significant difference.

## RESULTS

### Addition of exogenous IFN-γ or IL-4 decreases the Foxp3 expression induced by TGF-β

**Figure 1:**
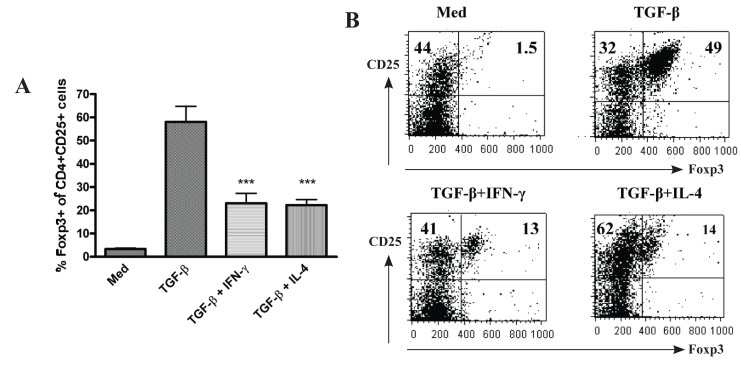
Exogenous IFN-γ or IL-4 diminishes the ability of TGF-β to induce CD4^+^CD25^-^ cells to express Foxp3. Splenic naïve CD4^+^CD25^-^ cells isolated from C57BL/6 mice were stimulated with immobilized anti-CD3, soluble anti-CD28 and IL-2 ± TGF-β with or without exogenous IFN-γ or IL-4 for four days, and examined by flow cytometry. The percentage of activated CD4^+^CD25^+^ cells expressing Foxp3 is shown and values indicate the mean ± SEM of three separate experiments. *P* values were calculated by Student *t* test and indicate significant effects of IFN-γ or IL-4 on TGF-β treated cells (***p<0.001). **(A)**, Scatter plots are gated on CD4, and the percentage of cells expressing both CD25 and Foxp3 is shown in the upper left and right quadrant. The example shown is representative of three separate experiments **(B)**.

As described previously by us and others, naïve CD4^+^CD25^-^ T cells activated with anti-CD3/CD28 in the presence IL-2 and TGF-β become CD4^+^CD25^+^ and more than 50% CD25^+^ cells have been converted into Foxp3^+^ cells (13, 23, Fig. 1). TGF-β plays a unique role in the induction of Foxp3 expression and development of suppressive activity since TGF-β failed to induce CD4^+^CD25^-^ cells from TGF-β receptor II dominate mice to express Foxp3 (data not shown). As IL-2 facilitates the induction of Foxp3 expression and IL-6 diminishes Foxp3 expression induced by TGF-β (20, 21, 32), it is possible other cytokines also affect the Foxp3 expression in CD4^+^CD25^+^ cells induced by TGF-β. We consider the possibility that cytokines which induce the differentiation of Th1 or Th2 cells may reduce the ability of TGF-β to induce Foxp3 since TGF-β suppresses Th1 and Th2 differentiation. As shown in Fig. 1, addition of exogenous IFN-γ or IL-4 markedly decreased the ability of TGF-β to induce Foxp3. These data indicate that different cytokines affect the differentiation of CD4^+^ cells and induction of TGF-β-iTreg cells needs specific cytokine profiles in addition to TCR engagement.

### Neutralization of IFN-γ or IL-4 markedly enhances the ability of TGF-β to induce Foxp3 expression and develop suppressive activity

**Figure 2:**
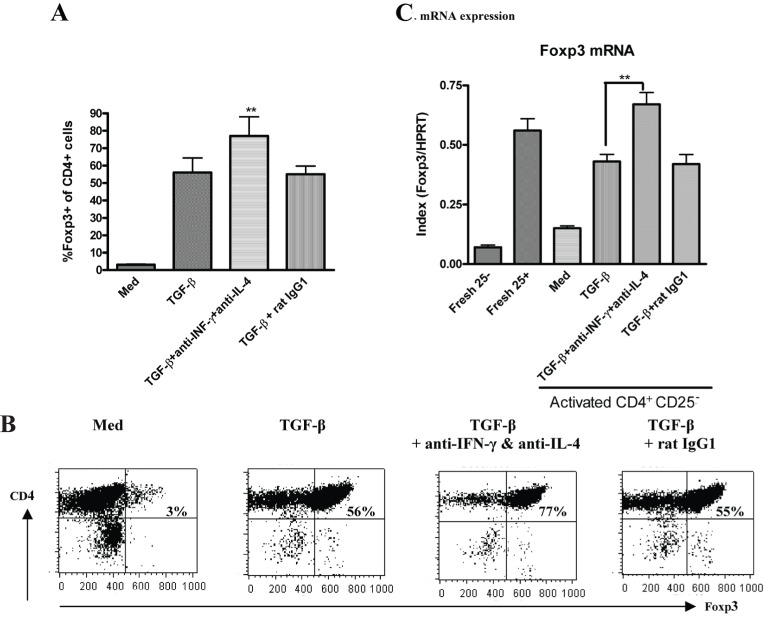
Neutralization of IFN-γ and IL-4 significantly increases the ability of TGF-β to induce naïve CD4^+^CD25^-^ cells to express Foxp3. Splenic CD4^+^CD25^-^ cells isolated from C57BL/6 mice were stimulated with immobilized anti-CD3, soluble anti-CD28 and IL-2 ± TGF-β with or without anti-IFN-γ and IL-4 or control rat IgG1 for four days. Foxp3 expression among CD4^+^ cells was determined by FACS analysis as described in Figure 1. **(A)**, Values indicate the mean ± SEM of four separate experiments. *P* values indicate significant effects of anti-IFN-γ and anti-IL-4 on TGF-β treated cells compared to control IgG (**p<0.01). **(B)**, The percentage of CD4^+^Foxp3^+^ cells is shown in the upper right quadrant and is representative of four independent experiments. **(C)**, Values indicate the mean ± SEM for four separate experiments of Foxp3 mRNA semiquantitative expression on the different groups of cells (**p<0.01).

Since the addition of exogenous IFN-γ or IL-4 markedly decreased the ability of TGF-β to induce Foxp3, we next considered whether neutralization of IFN-γ or IL-4 can increase the ability of TGF-β to induce Foxp3. Accordingly, IL-2 + TGF-β induced 56% of CD4^+^CD25^+^ cells to express Foxp3, addition of anti-IFN-γ or anti-IL-4 slightly increased Foxp3 expression in IL-2/TGF-β-induced CD4^+^CD25^+^ cells (about 64%, data not shown), however, Foxp3 expression was significantly unregulated to 77% when both anti-IFN-γ and anti-IL-4 were simultaneously added to cultures (Fig. 2A & B). Consistently, neutralization of anti-IFN-γ and anti-IL-4 also enhanced the Foxp3 mRNA expression of IL-2/TGF-β-induced CD4^+^CD25^+^ cells (Fig. 2C).

**Figure 3:**
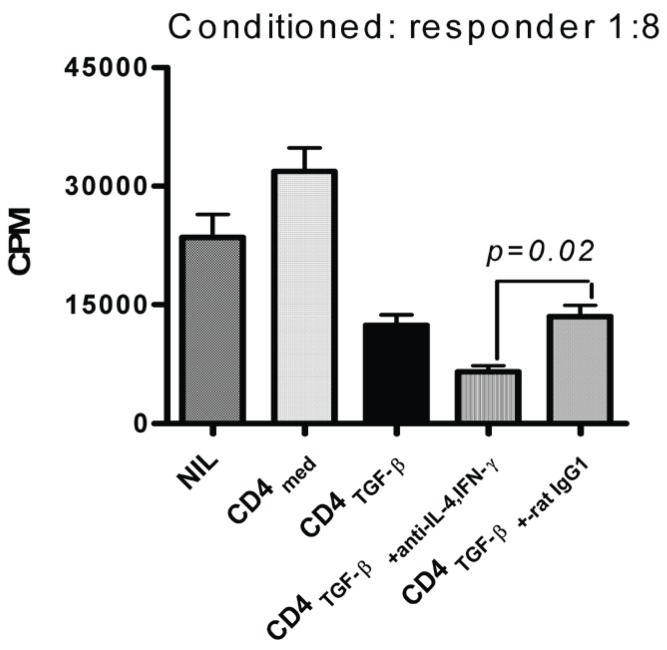
Neutralization of IFN-γ and IL-4 significantly increases the suppressive activity of TGF-β-induced Treg cells. Conditioned CD4^+^ cells were similarly generated as described in Figure 2. Suppression was assayed using anti-CD3-stimulated cells as described in *Materials and Methods*. The ratios of CD4_med_ (without TGF-β) or CD4_TGF-β_ to responder T cells are shown (1:8). This result is representative of four independent experiments. NIL indicates no added cells. *P* values indicate significant effects of anti-IFN-γ and anti-IL-4 on TGF-β treated cells compared to control IgG (*p=0.02*).

We also assessed suppressive activities of CD4^+^CD25^+^ cells induced by IL-2 and TGF-β with or without anti-IFN-γ and anti-IL-4 or control IgG. The experiment shown in Fig. 3 reveals that the suppressive activity of iTreg induced by IL-2 and TGF-β in the presence of both anti-IFN-γ and anti-IL-4 antibodies is significantly greater than in the absence of antibodies. These data suggest that neutralization of IFN-γ and IL-4 not only increases the Foxp3 expression in CD4^+^CD25^+^ cells induced by IL-2 and TGF-β, but also enhances the suppressive activity of these cells.

## DISCUSSION

The present study evaluates the ability of neutralization of IFN-γ and IL-4 to induce and enhance suppressive functions of TGF-β-induced CD4^+^CD25^+^ cells. We show that TCR engagement in conjunction with TGF-β stimulation can up-regulate Foxp3 expression and induce the suppressive activity of iTreg cells from peripheral CD4^+^CD25^-^ T cell progenitors. Whereas we demonstrate that exogenous IFN-γ or IL-4 diminishes the ability of TGF-β to induce CD4^+^CD25^-^ cells to express Foxp3, we also observed that the neutralization of IFN-γ and IL-4 markedly promote Foxp3 expression by TGF-β-induced iTreg cells and significantly enhances the suppressive activity of these cells.

Although TGF-β suppresses the differentiation of Th1 and Th2 cells, the existence of IFN-γ or IL-4, which respectively promotes the differentiation of Th1 or Th2 cells (33), interrupts the differentiation of Foxp3^+^ Treg cells initiated by TGF-β. It is similar that addition of IFN-γ or IL-4 inhibits the differentiation of Th17 cells induced by combination of IL-6 and TGF-β (25-27). IL-2 fails to overcome the effect of IFN-γ or IL-4 on TGF-β-induced Foxp3 expression in CD4^+^ cells although IL-2 critically involves in the development and expansion of TGF-β-induced Foxp3^+^ Tregs (23, 24).

The mechanism(s) by which IFN-γ or IL-4 affects the ability of TGF-β to induce Fopx3^+^ Treg cells remains unclear. Although cytokines promoting the differentiation of Th1, Th2, Th17 or Treg cells are known to antagonize each other (34), it is also possible that they have some synergizing role in promoting the differentiation of distinct CD4^+^ subsets. Others have reported while IL-4 favors Th2 differentiation, combination of IL-4 and TGF-β resulted in generation of Th1 cells (35). The importance of IFN-γ in regulating TGF-β production was further confirmed in the study showing that CD4^+^ cells from IFN-γ^-/-^ mice produced more TGF-β compared to wild type mice (35).

It is likely the neutralization of IFN-γ or IL-4 enhances Foxp3 expression in CD4^+^CD25^+^ cells induced by TGF-β. As shown in Fig. 1 and reported previously, only 50% of TGF-β-induced CD4^+^CD25^+^ cells express Foxp3 (23). This implies that fully half of, the TGF-β-induced CD4^+^CD25^+^ cells do not express Foxp3 and may represent CD4^+^ effector cells. In order to reliably generate induced Treg cells *ex vivo*, it will be important to increase CD25^+^Foxp3^+^ and decrease CD25^+^Foxp3^-^ cells since suppressive activity is closely associated with Foxp3 expression (23). This study establishes a new protocol that will improve the ability of TGF-β to induce iTreg cells *ex vivo*, thereby increasing the likelihood that manipulation of TGF-β-induced iTreg cells may provide a novel therapeutic strategy for the treatment of autoimmune diseases and other chronic inflammatory diseases.

## CONFLICT OF INTEREST

The authors declare that no conflicting interests exist.
